# Bioactivity of volatile organic compounds by *Aureobasidium* species against gray mold of tomato and table grape

**DOI:** 10.1007/s11274-020-02947-7

**Published:** 2020-10-17

**Authors:** A. Di Francesco, J. Zajc, N. Gunde-Cimerman, E. Aprea, F. Gasperi, N. Placì, F. Caruso, E. Baraldi

**Affiliations:** 1 CRIOF–Department of Agricultural Sciences, University of Bologna, Via Gandolfi, 19, 40057 Cadriano, Bologna Italy; 2 Department of Agricultural and Food Sciences, University of Bologna, Viale Fanin, 42, 40127 Bologna, Italy; 3 Plant Protection Department, Agricultural Institute of Slovenia, Hacquetova ulica 17, 1000 Ljubljana, Slovenia; 4 Department of Biology, Biotechnical Faculty, University of Ljubljana, Jamnikarjeva 101, 1000 Ljubljana, Slovenia; 5 Center Agriculture Food Environment, University of Trento/Fondazione Edmund Mach, 38010 San Michele all’Adige, TN Italy; 6 Research and Innovation Centre, Fondazione Edmund Mach, via Mach 1, 38010 San Michele all’ Adige, Trento Italy

**Keywords:** Antibiosis, *Botrytis cinerea*, Postharvest, VOCs

## Abstract

*Aureobasidium* strains isolated from diverse unconventional environments belonging to the species *A. pullulans*, *A. melanogenum*, and *A. subglaciale* were evaluated for Volatile Organic Compounds (VOCs) production as a part of their modes of action against *Botrytis cinerea* of tomato and table grape. By in vitro assay, VOCs generated by the antagonists belonging to the species *A. subglaciale* showed the highest inhibition percentage of the pathogen mycelial growth (65.4%). In vivo tests were conducted with tomatoes and grapes artificially inoculated with *B. cinerea* conidial suspension, and exposed to VOCs emitted by the most efficient antagonists of each species (AP1, AM10, AS14) showing that VOCs of AP1 (*A. pullulans*) reduced the incidence by 67%, partially confirmed by the in vitro results. Conversely, on table grape, VOCs produced by all the strains did not control the fungal incidence but were only reducing the infection severity (< 44.4% by *A. pullulans*; < 30.5% by *A. melanogenum*, and *A. subglaciale*). Solid-phase microextraction (SPME) and subsequent gas chromatography coupled to mass spectrometry identified ethanol, 3-methyl-1-butanol, 2-methyl-1-propanol as the most produced VOCs. However, there were differences in the amounts of produced VOCs as well as in their repertoire. The EC_50_ values of VOCs for reduction of mycelial growth of *B. cinerea* uncovered 3-methyl-1-butanol as the most effective compound. The study demonstrated that the production and the efficacy of VOCs by *Aureobasidium* could be directly related to the specific species and pathosystem and uncovers new possibilities for searching more efficient VOCs producing strains in unconventional habitats other than plants.

## Introduction

*Aureobasidium pullulans* (de Bary) is a highly adaptable polymorphic species characterized by great phenotypic plasticity and ubiquitous ecology. The species complex comprised four varieties until the large genomic differences and substantial physiological differences prompted the separation into four separate species: *A. pullulans*, *A. melanogenum*, *A. subglaciale*, and *A. namibiae*. These species display different patterns of melanisation, different temperature growth limits and salt tolerance (Gostinčar et al. 2014), various biofilm forming capacity and differences in repertoire of enzymatic activities (Zajc et al. 2019; Zajc et al. 2020). Importantly, the strains linked to human infections and able to grow at 37 °C all clustered together in a well-defined entity, *A. melanogenum*, preventing the inclusion of any potentially pathogenic strains in biotechnological applications, including biocontrol. Whit regard to *A. namibiae*, the species name was based on a single isolate from Namib Desert marble (Zalar et al. 2008), an extreme environment that could represent an interesting source for new microorganisms characterized by interesting and new antimicrobial metabolites. On the other hand, the species *A. pullulans* is recognized as one of the most promising biocontrol agents used in plant protection, in particular against postharvest diseases (Di Francesco et al. 2018; Di Francesco et al. 2020a; Zhang et al. 2010), while *A. subglaciale* is the least studied species in the field of crop defense, without any recognized biocontrol potential.

Although *A. pullulans* is mostly found associated with phyllosphere and carposphere of various plants (Bozoudi and Tsaltas 2018), it populates also diverse extreme habitats, from hypersaline water of salterns, glacial ice, polluted water, frozen and salt-preserved food, household surfaces and house dust, synthetic polymers and aviation fuel tanks (Gostinčar et al. 2019). The species *A. melanogenum* has been isolated mainly from oligotrophic and aqueous environments, while psychrotolerant *A. subglaciale*, able to grows at 4 °C, occurs mainly in glacial habitats (Gostinčar et al. 2014).

The differences between these species could be also deepened through the study of the mechanisms of action against fungal pathogens. Antibiosis is considered a biological process by which antagonists produce substances that inhibit or kill potential pathogens in close proximity (Di Francesco et al. 2015). Antibiosis can occur via volatile, low-molecular weight carbon-based organic compounds, derived from a biosynthetic pathway (Gotor-Vila et al. 2017). Many Volatile Organic Compounds (VOCs) show a fungistatic activity, in particular against postharvest fungal diseases of fruit (Di Francesco et al. 2015; Gotor-Vila et al. 2017). Identification of active secondary VOCs metabolites enables their use in process-defined biofumigation or in active packaging, where there is no direct contact between BCAs and the pathogens on food (Di Francesco et al. 2015).

Since volatile metabolites that inhibit the growth of fungal pathogens can also influence fruit/food matrix odor, taste, color, and texture, it is necessary to identify individual components. The solid-phase microextraction (SPME) coupled to GC–MS is an established method for extraction and identification of specific fungal VOCs. Fungal VOCs identified so far with SPME–GC–MS mainly belong to the alcohol class (Di Francesco et al. 2015), to the esters (Fialho et al. 2010), and aldehydes (Buzzini et al. 2003).

*Aureobasidium pullulans* is known to produce VOCs active against postharvest pathogens such as *Monilinia* spp. and *Botrytis cinerea* (Di Francesco et al. 2015, 2020a), at moderate concentrations, making them extremely attractive for postharvest diseases management. Nevertheless, until now no studies were conducted to uncover potential differences in volatilomes of different species of the former *A. pullulans* complex.

Thus, the objectives of the present study were (i) to characterize the main VOCs produced by *A. pullulans, A. subglaciale,* and *A. melanogenum* with the SPME/GC–MS; (ii) to evaluate the effectiveness of volatile metabolites produced by *Aureobasidium* spp. against *B. cinerea* by in vitro and in vivo assays; and (iii) to test the antifungal effect of pure main compounds of the selected volatiles on target pathogen in in vitro experiments.

## Materials and methods

### *Aureobasidium* strains

*Aureobasidium* strains reported in Table 1 were obtained from the Ex Culture Collection of the Infrastructural Centre Mycosmo (Department of Biology, Biotechnical Faculty, University of Ljubljana, Ljubljana, Slovenia), and the Westerdijk Fungal Biodiversity Institute (Utrecht, The Netherlands) and used for the present experiments. Strains were maintained on nutrient yeast dextrose agar (NYDA: 8 g L^−1^ of nutrient broth, 5 g L^−1^ of yeast extract, 10 g L^−1^ of dextrose and 15 g L^−1^ of technical agar, Oxoid, Basingstoke, UK) and stored at 4 °C until use. Two days before the experiments, each antagonist was grown on NYDA at 25 °C, and yeast cells were resuspended in sterile distilled water containing 0.05% (v/v) Tween-80 (Sigma-Aldrich, St. Louis, MO, USA) then adjusted to a final concentration of 10^8^ Colony Forming Unit (CFU) mL^−1^ (Di Francesco et al. 2017).

**Table 1:** List of strains used in this study

Species	Culture collection strain number	Present study number	Isolation habitat	Sampling site location
*Aureobasidium pullulans*	EXF-6519	AP1	Felt of a metal roof tile	Slovenia (Mengeš)
	EXF-10507	AP2	Marble block surface	Italy (Messina)
	EXF-10629	AP3	Car petrol reservoir	Slovenia (Jezero)
	EXF-10650	AP4	Acrylic painting	Slovenia (Solkan)
	EXF-10751	AP5	Cloud water	France
*Aureobasidium melanogenum*	EXF-3378/CBS 110374	AM6	Public fountain	Thailand
	EXF-3397	AM7	Endoperitoneal fluid	Greece
	EXF-8016	AM8	Bathroom, between faucet and sink	nd
	EXF-8429	AM9	Tap water	Slovenia
	EXF-11028	AM10	Proteus anguinus aquarium water	Slovenia, Ljubljana
*Aureobasidium subglaciale*	EXF-2481/CBS 123387	AS11	Subglacial ice	Arctic; Svalbard, Ny Alesund
	EXF-4632	AS12	From decaying leaves of *Convallaria*	Slovenia
	EXF-2425	AS13	Subglacial ice	Arctic; Svalbard, Ny Alesund
	EXF-2428	AS14	Subglacial ice	Arctic; Svalbard, Ny Alesund
	EXF-2450	AS15	Glacial ice	Arctic; Svalbard, Ny Alesund

### Pathogen

The isolate of *B. cinerea* (Bc1), originally isolated from a rotted peach, belongs to CRIOF-DipSA collection. The isolate was grown on agar (60 g L^−1^ of oat meal, 10 g L^−1^ of sodium nitrate, 30 g L^−1^ of sucrose, 12 g L^−1^ of agar) and incubated at 25 °C for 10 days and verified for its virulence on tomato and grape. Pathogen conidia were collected and suspended in sterile distilled water containing 0.05% (v/v) Tween 80 and the suspension was adjusted to 10^5^ conidia mL^−1^, by counting the conidia under the microscope by using a haemocytometer (Di Francesco et al. 2020b).

### Tomato and table grape

Tomatoes *cv* “Datterini” (*Solanum lycopersicum*, L.) were harvested from an organic orchard located in Cesena (Italy) at commercial maturity and were immediately used.

Table grapes *cv* ‘Vittoria’ were harvested in experimental orchards of Bologna University located in Altedo (Bologna-Italy) (44° 39′ 42.9″ N 11° 29′ 29.0″ E) (Lohmar 1988). After harvest, fruits with no visible wounds and rots, homogenous in size and quality (soluble solid content, hardness, color), were disinfected by 1 min immersion in hypochlorite 0.1% (w/v), rinsed with tap water and air dried at room temperature. Soon after disinfection, fruits were wounded with a sterile nail (3 × 3×3 mm) on the equator (one wound per fruit) and inoculated with pathogen conidial suspension (15 µL).

### Volatile organic compounds analysis

VOCs analysis was carried out by using SPME/GC‐MS. Glass vials containing 10 mL of NYDA were inoculated with 100 μL of each yeast strain suspension (10^8^ CFU mL^−1^), and the media alone represented the blank reference. Vials were incubated at 25 °C for 48 h and soon after the VOCs were extracted and preconcentrated by solid phase microextraction (SPME) using a 2 cm PDMS/DVB/CAR fiber (Supelco, Milan, Italy). The fibers were exposed for 30 min, after VOCs were desorbed into the GC injector port for 5 min at 250 °C. These operations were performed by an automatic system (CTC Analysis AG, Zwingen, Switzerland). The chromatographic separation was performed via an HP‐Innowax fused‐silica capillary column (30 m, 0.32 mm i.d., 0.5 μm film thickness: Agilent Technologies, Santa Clara, California). The GC oven temperature program was as follows: 40 °C for 3 min, raised from 40 to 220 at 4 °C min^−1^, 220 °C for 1 min, raised from 220 to 250 at 10 °C min^−1^ and 250 °C for 1 min. Helium was used as carrier gas with a constant column flow rate of 1.5·mL min^−1^. The transfer line temperature was maintained constant at 220 °C. Upon exiting the column, compounds were ionized via electron impact at 70 eV and detected with a quadrupole mass spectrometer in the range of a mass/charge ratio (*m*/*z*) from 30 to 300. Chromatograms were processed and analysed by TurboMass v. 5.1 software (Perkin Elmer, Norwalk, CT). Compounds identification was achieved by comparing the spectra with the NIST Standard Reference Database (NIST2014) and by linear retention indices (LRI) calculated under the same chromatographic conditions, injecting C7–C30 *n*‐alkane series (Supelco, Milan, Italy). The relative VOC content of each sample was reported as absolute peak area (Aprea et al. 2012). The sample unit was represented by four vials per each yeast strain and the experiment was conducted once.

### In vitro assay

Effectiveness exerted by VOCs produced by *Aureobasidium* strains belonging to three different species was assayed by the double Petri dish assay (Di Francesco et al. 2020a). VOCs were tested against mycelium growth of *B. cinerea* and for this purpose, NYDA plates were inoculated by spreading 100 μL of each antagonist cell suspension (10^8^ CFU mL^−1^) incubated at 25 °C for 2 days. The control consisted of plates without the antagonist. The sample unit was represented by ten plates (replicates) for the control and each *Aureobasidium* spp. strain. The lid of the plates was replaced by a base plate of MEA (Malt Extract Agar, 50 g L^−1^, Oxoid, UK), inoculated in the middle with a mycelium plug (6 mm of diameter) of the pathogen. The two base plates were sealed with a double layer of Parafilm and incubated at 25 °C for 5 days. The sample unit was represented by ten plates (replicates) for the control and each *Aureobasidium* strain interaction. The control consisted of plates without the antagonist interaction. The experiment was conducted twice. The inhibition rate of mycelial growth was calculated using the equation (Chen and Dai 2012):

$$\% \, {\text{of inhibition}} = \frac{{\left( {{\text{d}}1 - {\text{d}}2} \right)}}{{{\text{d}}1}} \times 100$$

where (%) is the percent of inhibition of pathogen mycelial growth; d1 represents the control colony diameter (mm); d2 represents the treated colony diameter (mm).

### In vivo assays

The antagonistic activity of VOCs produced by the most efficient *Aureobasidium* strains of each tested species (AP3, AM10, AS14) was tested for controlling gray mold symptoms in fruits. Tomatoes (20 fruits) and grapes (20 fruits) were placed in sterile glass boxes (24 × 18 × 8 cm, L × W × H) with a thin layer of NYDA (120 mL) positioned at the bottom and inoculated by spreading 500 μL of each yeast strain suspension (10^8^ CFU mL^−1^) and incubated at 25 °C for 2 days. Fruits were positioned on a sterile grid to separate them from the bottom substrate and avoid the direct contact and possible contaminations (Di Francesco et al. 2020a). Each fruit was wounded and inoculated with 15 μL of suspension of *B. cinerea* (10^5^ conidia mL^−1^). The boxes were closed with plastic lid and immediately sealed with Parafilm. The control consisted of inoculated fruit placed in boxes without yeast suspensions. The boxes containing inoculated fruit were kept at 20 °C. The percentage of rotten fruits and the lesion diameters were measured after 5 days of incubation. The sample unit was represented by two boxes per each yeast strain. The experiment was conducted twice.

### Synthetic volatile organic compound effect on *Botrytis cinerea* mycelial growth

Using a microsyringe, pure 3-methyl-1-butanol, 2-methyl-1-propanol, and ethanol (Sigma–Aldrich, St. Louis, MO) were placed in quantity of 25, 50, and 100 μL on a filter paper (Whatmann No. 1, 90 mm diameter) positioned inside the cover of a MEA dish previously inoculated in the middle with 6 mm pathogen mycelial plug. The aliquots of pure compounds introduced in the Petri dishes corresponded to 2.25, 1.12, and 0.56 mL L^−1^ headspace, as described by Di Francesco et al. (2015). The dishes were immediately closed, sealed with Parafilm and incubated at 25 °C. The activity of each pure compound against mycelial growth was evaluated after 5 days of incubation. In the control, pure compounds were substituted by equivalent amounts of distilled water. Ten plates for each VOC concentration represented the sample unit and the experiment was conducted twice. EC_50_ values were calculated as the headspace concentrations (mL L^−1^) that inhibited pathogen mycelial growth by 50% compared with the control.

### Statistical analysis

Data were analysed by one-way analysis of variance (ANOVA). Statistical comparison of means was carried out by using Tukey’s HSD Test (α = 0.05). All analyses were performed with Mini Tab 16. The EC_50_ values of each compound were calculated using the probit analysis applied to the percentage of inhibition of the pathogen mycelial growth (Lesaffre and Molenberghs 1991).

## Results

### Volatile organic compounds analysis

The headspace VOCs of *Aureobasidium* strains were isolated and analyzed by SPME/GC‐MS. Absolute peak areas (AA) of detected and identified compounds are shown in Table 2.

**Table 2:** Analyses of volatile organic compounds (VOCs) produced by *Aureobasidium* spp. with HS-SMPE and GC–MS

Compound	RT	Absolute areas (AA).		
		*Aureobasidium* spp.		
		*Pullulans*	*Melanogenum*	*Subglaciale*
Ethyl acetate	2.51	1.10E+06a	1.85E+06b	4.65E+06c
Ethanol	3.20	5.39E+08c	4.22E+08b	3.18E+08a
2-Methyl-1-propanol	7.33	1.66E+07b	1.93E+07c	1.18E+07a
Isoamyl acetate	8.13	8.53E+04a	1.53E+05b	9.59E+04a
3-Methyl-1-butanol	10.74	5.76E+07b	7.52E+07c	3.66E+07a
2-Methyl-2-butenol	14.05	1.07E+05b	4.87E+04a	2.00E+05c
Methyl benzoate	21.86	2.79E+04b	8.26E+03a	2.69E+04b
Butyrolactone	21.97	3.96E+04c	0.00E+00a	3.21E+04b
3-Ethylbenzaldehyde	23.85	1.32E+03c	0.00E+00a	7.66E+02b
γ-Cadinene	24.93	1.98E+02c	0.00E+00a	1.00E+02b
α-Phenylethanol	26.16	3.58E+03a	7.34E+03c	4.50E+03b
p-Acetylethylbenzene	26.55	1.21E+03c	3.51E+02b	0.00E+00a
(Z)-Cinnamaldehyde	26.60	1.36E+02a	3.74E+02b	4.63E+02c
Ethyl tetradecanoate	30.96	4.27E+04c	8.66E+03a	1.56E+04b

DVB/CAR/PDMS SPME fiber was used. The values represent the average of the same compound produced by 5 strains (each analysed by four vials) that belong to the same species (*A. pullulans, A. melanogenum, A. subglaciale*). Different letters represent significant differences among the species for each compound according to *Tukey’s* HSD Test (α = 0.05)

**Fig. 1:**
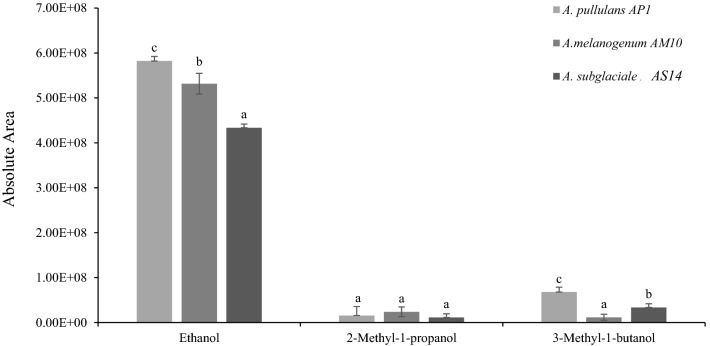
Principal Volatile Organic Compounds (VOCs) produced by *Aureobasidium* spp. strains AP1 (*A. pullulans*), AM10 *(A. melanogenum*), AS14 (*A. subglaciale*) and detected with HS-SMPE and GC–MS gas phase. DVB/CAR/PDMS SPME fiber was used. Each value is the mean ± standard deviation of four replicates for each strain. Different letters indicate significant differences according to Tukey’s HSD Test (α = 0.05)

Compounds belonging to the class of alcohols dominated production by all the three *Aureobasidium* species without significant differences between them, in particular ethanol, 3-methyl-1-butanol, and 2-methyl-1-propanol. The identification of these compounds was confirmed by comparison with standards and used for subsequent experiments. Among tested *Aureobasidium* species only *A. melanogenum* did not produce butyrolactone, 3-ethylbenzaldehyde, and γ-cadinene (Table 2). VOCs mainly produced by each strain belong to the species that showed more effectiveness against *B. cinerea* in previous experiments. The *A. pullulans* strain AP1 displayed a higher activity of target VOCs production with respect to *A. melanogenum* AM10 and *A. subglaciale* AS14 strains (Fig. 1) mainly for ethanol and 3-methyl-1-butanol.

### In vitro assay

**Fig. 2:**
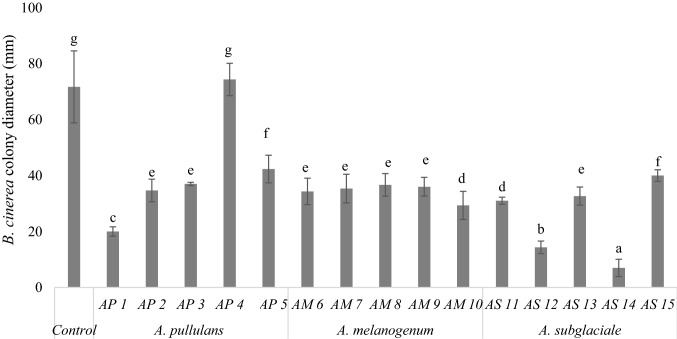
Effect of volatile compounds produced by *Aureobasidium* strains belonging to *A. pullulans, A. melanogenum*, and *A. subglaciale* species on the mycelial growth (mm) of *Botrytis cinerea.* Colony diameter (mm) was measured after 5 days at 25 °C. Each value is the mean of 10 plates (replicates) ± standard deviation. Different letters represent significant differences among the strains according to *Tukey’s* HSD Test (α = 0.05)

The antifungal effect of VOCs produced by *Aureobasidium* strains was tested on *B. cinerea* by the double Petri dish assay system (Di Francesco et al. 2015), avoiding any physical contact between the yeasts and the pathogen. The VOCs produced by all tested strains significantly inhibited *B. cinerea* mycelial growth, except for the *A. pullulans* strain AP4 (Fig. 2), indicating variability in the antagonistic activity among strains of *A. pullulans*. *Aureobasidium subglaciale* showed the highest inhibition percentage of the pathogen mycelial growth (65.4% on average), with the strains AS11 and AS14 reducing *B. cinerea* growth by 80% and 90.2%, respectively. Also *A. pullulans* AP1 strain showed high effectiveness (72.1%). On the other hand, the strains belonging to *A. melanogenum* showed low variability of VOCs effect on *B. cinerea* with an average of 51.9% of inhibition.

### In vivo assays

**Fig. 3:**
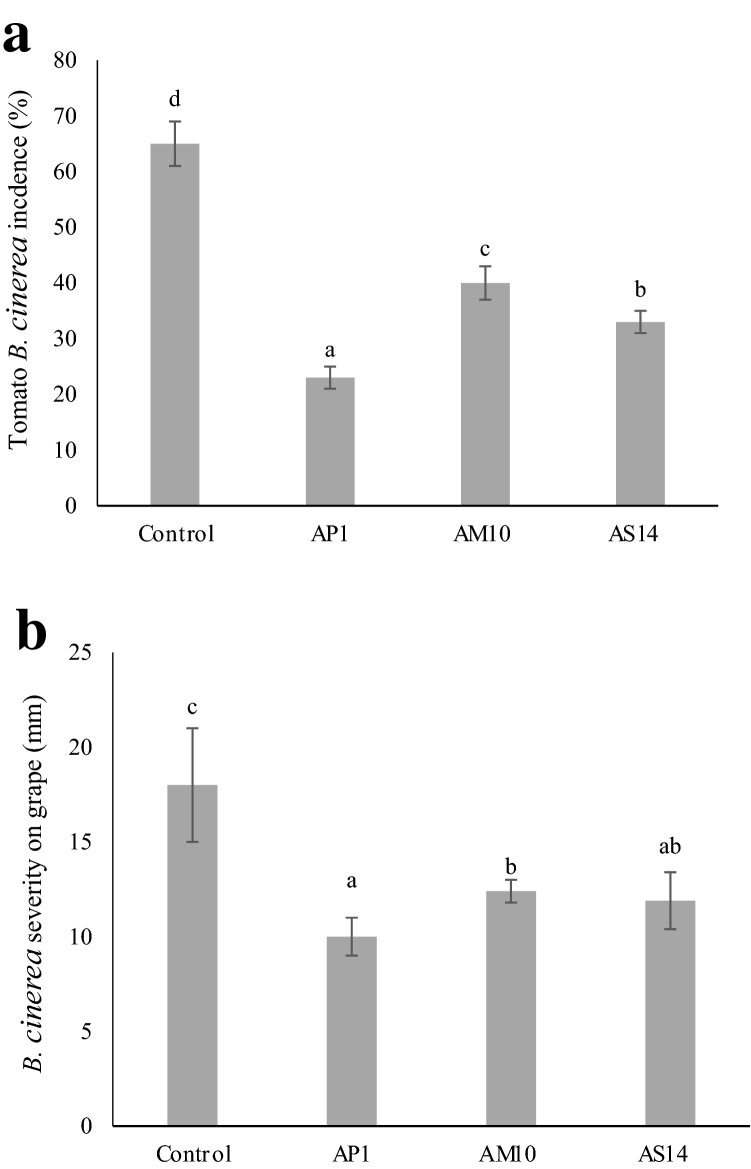
*In vivo* antagonistic effect of VOCs produced by *Aureobasidium* strains on *Botrytis cinerea* in tomato (**a**) (disease incidence— %) and table grape (**b**) (disease severity, indicated as the average of two diameter measurements—mm). Fruits were artificially inoculated with *B. cinerea* conidial suspension (10^5^ conidia mL^−1^) and incubated for 5 days at 20 °C and 85% RH. Control consisted of NYDA without yeast inoculation. Each value is the mean ± standard deviation of two replicates of 20 fruit each for tomato and grape, respectively. Different letters represent significant differences among the yeasts VOCs treatments according to *Tukey’s* HSD Test (α = 0.05)

**Fig. 4:**
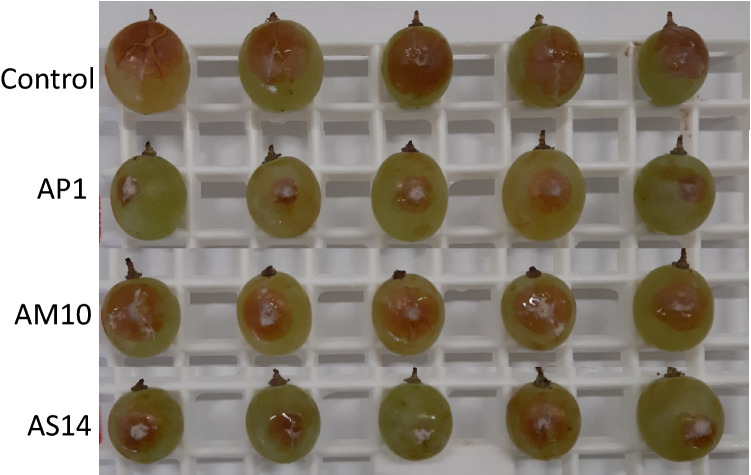
Effect of volatile organic compounds (VOCs) produced by AP1, AM10, AS14 strains, respectively *A. pullulans, A. melanogenum,* and *A. subglaciale*, on table grape artificially inoculated with *Botrytis cinerea* conidial suspension

The VOCs produced by the most effective strains AP1, AM10, AS14 were tested against *B. cinerea* on tomato and table grape (Fig. 3a, b). Regarding tomato, the fungal pathogen was better controlled by AP1 (*A. pullulans*) VOCs, showing 67% of incidence reduction. Strains AM10 (*A. melanogenum*) and AS14 (*A. subglaciale*) reduced fungal incidence of 38.4% and 49.2% respectively (Fig. 3a). On tomato, VOCs produced by the tested antagonist strains blocked the pathogen sporulation. When assayed on table grape, the VOCs of the tested strains did not reduce the fungal incidence which was really high (up to 90%, data not shown) but significantly reduced symptom severity: *A. pullulans* AP1 for 44.4%, and both *A. melanogenum* AM10 and *A. subglaciale* AS14 for 30.5% (Fig. 3b). On grape, VOCs produced by all strains stimulated the fungal sporulation (Fig. 4).

### Synthetic volatile compound effect on *Botrytis cinerea* mycelial growth

As commented above, the compounds 3-methyl-1-butanol, 2-methyl-1-propanol, and ethanol, which resulted as the main VOCs produced by *Aureobasidium* spp., were tested for their inhibitory activity on mycelial growth of *B. cinerea*. 3-methyl-1-butanol was the most effective compound, showing the lowest EC_50_ value (0.09 mL L^−1^). In comparison ethanol and 2-methyl-1-propanol showed the lowest antifungal activity against *B. cinerea* mycelial growth, with EC_50_ values ranging from 0.20 to 0.51 mL L^−1^, respectively (Table 3). Furthermore, no quality alterations (color, texture, odor) were detected on the VOCs treated fruits (data not reported).

**Table 3:** EC_50_ values (mL L^−1^) of synthetic volatile compounds mainly produced by *Aureobasidium* spp. strains evaluated on mycelial growth (Ø, mm) of *Botrytis cinerea* (*Bc1*)

	EC_50_ values (mL L ^− 1^)
Ethanol	0.51
3-Methyl 1 butanol	0.09
2 Methyl 1 propanol	0.20

The mycelial growth measurements were carried out after 5 days of incubation at 25 °C

## Discussion

The *Aureobasidium pullulans* species complex was characterized by high genetic and phenotypic variability. Gostinčar et al. (2014) elevated the varieties of the *A. pullulans* species to the level of separate and well defined species based on large differences in genomic data together with distinctive ecology and physiology: *A. pullulans, A. subglaciale, A. namibiae,* and *A. melanogenum*. As *A. melanogenum* is recognized as opportunistic human pathogen (Gostinčar et al. 2019), it should not be used as a biocontrol agent, while *A. pullulans* is one of the most extensively studied and used biocontrol agents, in particular for postharvest diseases (Di Francesco et al. 2018, 2020a; Zhang et al. 2010). *Aureobasidium subglaciale,* isolated so far only in some specialized environments and characterised by low temperatures and oligotrophic conditions, was recently proposed to be used in biocontrol of storage fungal diseases (Di Francesco et al. 2020b).

While VOCs production and bioactivity of *A. pullulans* have been studied in the past, no such studies were performed for *A. subglaciale* or even *A. melanogenum.* Also, the strains of *A. pullulans* studied and characterized for the biological control against crop diseases, were isolated from plants habitats such as fruit or leaf surfaces (Di Francesco et al. 2018; Mounir et al. 2007). Conversely, the present study considered the possible antagonistic activity of *A. subglaciale* and *A. melanogenum* strains isolated from non-plant related environment. Although such strains may not have been tested with respect to their volatilomes, some of them were tested with respect to the antagonistic activity.

Fourteen out of fifteen strains of the three species produced VOCs that were shown to be active in vitro against gray mold by reducing the colony growth of *B. cinerea.* However, there were substantial differences in the effectiveness among the three species and even among individual strains of *A. pullulans* and *A. subglaciale.* Among *A. subglaciale,* the strains AS12 and AS14 reduced growth of *B. cinerea* by 80% and 92.2% respectively. Altogether this species showed the highest average inhibition (65.4%). In the case of *A. pullulans*, the strain AP1 exhibited 72.1% effectiveness, while AP4 did not inhibit at all the growth of *B. cinerea,* indicating a high diversity in efficiency in both *A. pullulans* and *A. subglaciale*. On the contrary, different *A. melanogenum* strains showed similar effects against *B. cinerea*, unregarding their isolation ecology.

Recently, it was shown that strains of *A. pullulans*, albeit possessing potent antagonistic activity against various plant pathogenic fungi, exhibit substantial differences in aspects linked to the mechanism of action underneath their antagonistic activity, for instance in enzymatic repertoire and the enzymatic activities, siderophore production, and tolerance to stress (Zajc et al. 2020). Strains of *A. pullulans* used in this study originated from different extreme habitats such as cloud water, roof tile, marble rock surface, painting, and even petrol reservoir. The genome resequencing of fifty strains of *A. pullulans* from diverse habitats confirmed its generalist nature and homogenous population structure due to high recombination within the species, without any observed specialization to a particular habitat or geographic location (Gostinčar et al. 2019). This suggests that potent antagonistic strains can be found also in habitats not associated with plants.

The best in vitro performing strains (AP1, AM10, and AS14) showed different performances in vivo. *Aureobasidium pullulans* (AP1) appeared the most effective for controlling *B. cinerea* as it reduced gray mold incidence on tomato and severity on table grape. However, *A. subglaciale* (AS14) that showed the highest efficacy in reducing *B. cinerea* colony growth in in vitro assay, performed worse than *A. pullulans* in vivo. This phenomenon is not uncommon and was also observed in various previous studies (Chen et al. 2018). Conversely, Di Francesco et al. (2015) showed how *A. pullulans* strains L1 and L8 were more effective in the control of *B. cinerea* on apple than in inhibiting fungal conidial germination, displaying a reduction of the pathogen lesion diameter of 88.9% and 94.4% respectively. These results confirmed how VOCs produced by different yeast strains, also belonging to the same species, can have a different effect on a target pathogen and host.

*Aureobasidium subglaciale* has a much more restricted distribution than *A. pullulans* as it was mainly found in subglacial ice in the Arctics (Zalar et al. 2008), and as epiphyte on moss (Kachalkin 2010). It tolerates high gamma irradiation, UV light, heavy metal ions (Liu et al. 2017), and low growth temperatures (Gostinčar et al. 2014), making it interesting for postharvest biocontrol applications. Mestre et al. showed biocontrol potential of psychrophilic yeasts from Patagonia (Argentina) against some plant pathogens in cold-temperate regions (Mestre et al. 2011) or during low- temperature storage of fruit and vegetables. Our previous study showed its potential in biocontrol via non-volatile compounds (Di Francesco et al. 2020b).

*Aureobasidium melanogenum* VOCs displayed the lowest effectiveness against *B. cinerea*. However, *A. melanogenum* was recognized as an opportunistic human pathogen (Gostinčar et al. 2014), so its use in biological control due to the potential health risk is considered inappropriate. Importantly, given that this species was only recently separated from *A. pullulans*, a phylogenetic analysis of the twenty *A. pullulans* strains used so far in biocontrol confirmed that only *A. pullulans* strains (Zajc et al. 2020) were used in extensive postharvest disease control studies (Di Francesco et al. 2018; Parafati et al. 2015).

Interestingly, our study highlighted that VOCs produced by all tested strains reduced the gray mold severity but at the same time stimulated the *B. cinerea* sporulation on table grapes. Previous studies reported that dipping the fruits in diluted ethanol solution successfully prevented grey mold on grapes (Dao and Dantigny 2011; Gabler et al. 2004; Lichter et al. 2002; Romanazzi et al. 2007) and also demonstrated that ethanol fumigation controlled grey mold as well as or even better than SO_2_ (Lurie et al. 2006). The reason for enhanced sporulation of *B. cinerea* in our experimental setup might be in other VOCs than ethanol. A possible explanation could be that some VOCs could stimulate grape ripening, modifying fruit chemical composition and stimulating pathogen sporulation. In fact, as fruit ripen, post-harvest fungal pathogens become more aggressive and quickly colonize fruit tissue (Alkan and Fortes 2015). A possible explanation for the differences in pathogen aggressiveness after the exposure to VOCs on tomato and grapes observed in our study could be related to different physiological characteristics: tomato being climacteric and grape non-climacteric fruit. This remains to be further investigated. Also, many of these VOCs identified and tested by in vitro assay against pathogens growth can have a different effect with respect to the in vivo tests, where the microorganism is involved in complex interactions with the fruit response, microbiome, and volatilome, as well as the environmental conditions including atmosphere surroundings (Eckert and Ratnayake 1994). In fact, VOCs can play a role in host selectivity of some pathogens. In order to understand the nature of VOCs produced by the different *Aureobasidium* species, the SPME-GC–MS technique was adopted. Detected VOCs mainly belonged to the alcohols and esters classes, similarly to other *A. pullulans* strains (Di Francesco et al. 2015). Ethanol, 2-methyl-1-propanol, and 3-methyl-1-butanol were the principal VOCs produced by all the tested strains and in particular by AP1 (*A. pullulans*), followed by AM10 (*A. melanogenum*), and AS12 (*A. subglaciale*). However, based on the EC_50_ values of each pure synthetic metabolite tested against *B. cinerea* mycelial growth, 3-methyl-1 butanol was the most efficient compound (0.08 mL L^−1^) whereas the most produced and at the same time the least active alcohol was ethanol (0.51 mL L^−1^). A hierarchical clustering of *Aureobasidium* species based on the types of VOCs, showed that *A. pullulans* and *A. subglaciale* cluster closer together, producing more or less the same repertoire of VOCs (data not reported). The only VOC produced by *A. pullulans*, but not by *A. subglaciale,* was p-acetylethylbenzene. Furthermore, *A. pullulans* produced higher amounts of 3-ethylbenzaldehyde, γ-cadinene, and ethyl tetradecanoate than *A. subglaciale*. These compounds might be responsible for higher effectiveness of *A. pullulans* against *B. cinerea*, but this speculation requires further investigation to be confirmed. *Aureobasidium melanogenum* produced a high concentration of each detected compounds except for butyrolactone, 3-ethylbenzaldehyde, and γ-cadinene.

VOCs have many effects that are beneficial, from promoting plant growth to antagonizing plant pathogens. For instance, detected lactones belong to the class of amino compounds containing lipids produced by microorganisms, plant-associated bacteria, and plant growth-promoting rhizobacteria (PGPRs) (Kanchiswamy et al. 2015). Furthermore, benzaldehydes are noted to be antibacterial compounds (Friedman et al. 2003), again suggesting potential antimicrobial activity of VOCs produced by *A. pullulans*.

In conclusion, VOCs produced by *Aurebasidium* spp. were effective in suppressing mycelial growth of *B.* *cinerea* particularly in in vitro assays and partially also in vivo. This study showed that 3-methyl-1-butanol, and 2-methyl-1-propanol are the VOCs used as biocontrol agents produced by *Aureobasidium* strains, previously reported in other yeasts and endophytic fungi (Mitchell et al. 2010; Strobel et al. 2001). Our study uncovered differences among the three species regarding the amounts of produced VOCs. VOCs dynamic is extremely complex to understand mainly due to their versatile ecology and evolution paths. However, the results showed that the three tested yeast species produced the same VOCs, whit an exception for *A. melanogenum* volatilome deficient on butyrolactone, 3-ethylbenzaldehyde, and γ-cadinene volatile compounds.

By representing a bioactive interface between the pathogens and other microorganisms, VOCs are useful for crop protection and sustainable agriculture perspectives (Corcuff et al. 2011). Biofumigation with VOCs opens new possibilities to control postharvest microbial decays of fruits as they avoid physical contact of a biopesticide with the product.
